# A Case Report of Diabetic Striatopathy: An Approach to Diagnosis Based on Clinical and Radiological Findings

**DOI:** 10.7759/cureus.25089

**Published:** 2022-05-17

**Authors:** Gyusik Park, Hassan N Kesserwani

**Affiliations:** 1 Neurology, University of Alabama at Birmingham School of Medicine, Birmingham, USA

**Keywords:** hyperkinetic movement disorder, hemichorea, hemiballismus, diabetes mellitus, diabetic striatopathy

## Abstract

Diabetic striatopathy (DS) is an acute hyperkinetic movement disorder characterized by hemiballismus-hemichorea (HBHC) due to nonketotic hyperglycemia. DS manifests a fascinating interplay between endocrinopathy (diabetes), striatal (putamen, caudate nucleus, globus pallidus) pathology, and a dramatic neurological movement disorder, HBHC. The striking hyperintensity on imaging modalities such as computed axial tomography (CT) scan of the brain and T1-weighted magnetic resonance imaging (MRI) of the brain can mislead the clinician to an erroneous diagnosis of a cerebral hemorrhage and/or ischemic infarct, especially in an acute setting. We present an acute case of DS and outline the natural history, semiology, typical radiological findings, and therapeutic options. With careful and thoughtful analysis, an accurate diagnosis can be exacted, sparing the patient unnecessary anxiety and medical costs.

## Introduction

Diabetic striatopathy (DS) is an intriguing acute hyperkinetic movement disorder secondary to nonketotic hyperglycemia. DS is a rare disorder with an estimated prevalence of 1 in 100,000, which is likely an underestimation due to unfamiliarity and missed diagnosis [1]. It is more prevalent in females, and the greatest risk factor is old age [2]. Its sine qua non is the development of hemiballismus-hemichorea (HBHC) associated with hyperintensity on T1-weighted magnetic resonance imaging (MRI) of the putamen, caudate nucleus, and globus pallidus in various combinations, with the putamen being most involved [3,4]. Imaging usually shows an absence of mass effect or contrast enhancement, indicating an intact blood-brain barrier. The internal capsule is normally spared [5]. The most likely pathology of DS involves myelin destruction caused by swollen reactive astrocytes called gemistocytes [6]. This hyperkinetic movement disorder is usually improved with judicious glucose control, sometimes necessitating treatment with anti-chorea agents such as dopamine antagonists (haloperidol), vesicular monoamine uptake (VMAT-2) inhibitors (tetrabenazine), or gamma-aminobutyric acid (GABA) agonists (clonazepam) [7]. In rare cases, deep brain stimulation (DBS) of the putamen, sub-thalamic nucleus, and/or globus pallidus internus may be necessary to control the HBHC [8,9].

## Case presentation

We present the case of 51-year-old man with poorly controlled diabetes mellitus and a two-month history of continuous and involuntary motion of the left upper and lower extremity, sparing the tongue, face, and the neck. These movements did not abate with sleep. His speech was unaffected, but the cadence and smoothness of his gait was interrupted by the incessant motion. His past medical history was significant for chronic hypertension and he had at least a 10-year history of diabetes mellitus with poor adherence to anti-diabetic medications. There was no family history of chorea, and he did not smoke cigarettes or drink alcohol. His medications included metformin, metoprolol, pravastatin, and valsartan.

On examination, his blood pressure was 133 mmHg systolic and 84 mmHg diastolic with a pulse of 76 beats per minute. His height was six feet with a weight of 175 pounds and a body mass index of 23.7 kg/m^2^. On initial inspection, his examination was notable for high amplitude involuntary dyskinesias of the axis (trunk), left upper extremity, and left lower extremity. Athetotic and torsional movements of the left shoulder, elbow, wrist, hip, and ankle were noted (Video 1).

**Video 1:** Video of the patient with diabetic striatopathy (DS) demonstrating the athetotic and torsional movements of the left shoulder, elbow, wrist, and ankle.

On gait examination, the patient’s left foot and lower leg jerked during the swing phase, making his gait irregular and unsteady. His speech was legible with normal volume, prosody, and fluency, but seemed mildly pressured. His extraocular muscles were intact with normal-amplitude eye motion in all directions without nystagmus. Pursuits were not interrupted. Pupils were briskly reactive to light with normal accommodation. The rest of the cranial nerve examination was normal, and it should be noted that no oro-bucco-lingual dyskinesia, blepharospasm, or jaw deviation dystonia was noted. Blinking frequency was also normal.

**Figure 1:**
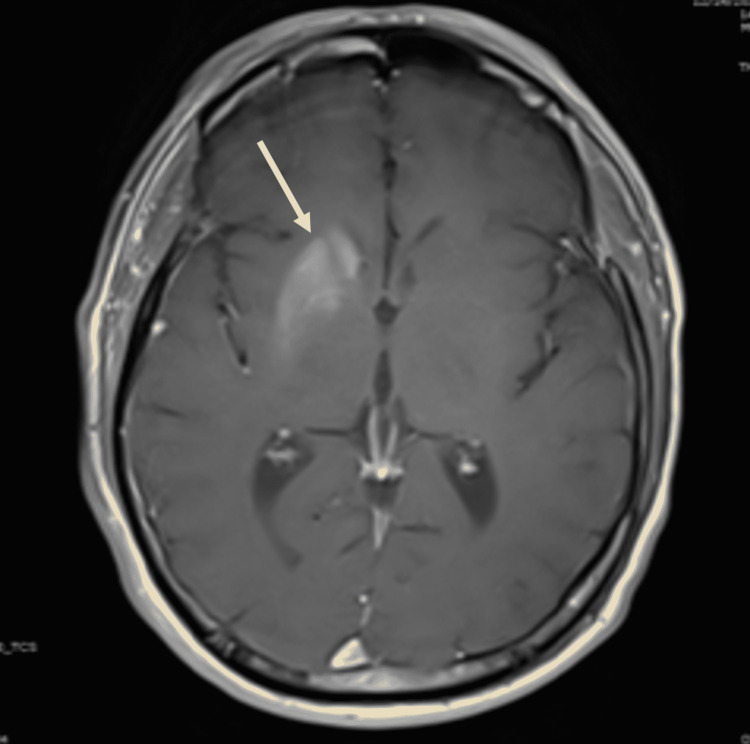
T1-weighted MRI of the brain showing hyperintensity in the right putamen and caudate nucleus (arrow).

Motor strength was preserved in the upper and lower extremities. Coordination examination with left finger-to-nose test and left heel-to-shin test demonstrated sinuous movements due to the choreoathetosis, but the target was reached. Patient’s deep tendon reflexes were lively throughout except for feeble bilateral ankle jerks. Sensory examination was generally preserved to small and large fiber modalities in the fingers and toes except for a diminished vibratory sense at the big toe bilaterally. A Romberg sign was absent. Extensor plantar responses were also absent. The patient’s glucose level was 301 mg/dL (normal range: 70-115 mg/dL), and his hemoglobin A1C was greater than 14% (normal range: 3.8-5.6%). The basic metabolic panel was otherwise normal. Serum and urine ketones were not measured. A T-1 weighted MRI study of the brain revealed a hyperintensity signal involving the right putamen and caudate nucleus (Figure 1).

Based upon the absence of a family history of chorea, the characteristic clinical history of chronic poorly controlled diabetes, and the characteristic MRI brain findings, a tentative diagnosis of DS was rendered. The patient was referred to an endocrinologist for better blood sugar management, and a trial of tetrabenazine at a dose of 12.5 mg twice daily was prescribed to reduce the amplitude and frequency of the disabling choreoathetosis. The patient demonstrated substantial improvement of the movement disorder in one month.

## Discussion

Acute HBHC is a defining clinical characteristic of DS. However, diagnosing DS is not always straightforward as there are many differential diagnoses of HBHC. Therefore, a structured approach should be taken to diagnose DS. We propose a two-fold method that involves running a differential diagnosis of HBHC and addressing the causes of MRI T1 hyperintensity of the striatum.

The possible causes of HBHC include metabolic, infectious, inflammatory, iatrogenic, autoimmune, and neurogenetic/neurodegenerative etiologies [10]. While the list is exhaustive, the clinical presentation and the associated laboratory findings should help narrow the differential diagnosis to a handful of causes. Common clinical presentations of DS include HBHC involving unilateral limbs, progression from upper to lower extremities, worsening of HBHC during nervousness, and suppression of HBHC during sleep [7]. A study involving 176 patients with DS showed an average blood glucose level of 414 mg/dL and hemoglobin A1C of 13.1% [7]. In our case, the patient’s acute onset of unremitting unilateral HBHC and the hemoglobin A1C of greater than 14% raised suspicion for DS. The acute nature of his symptoms and the lack of fever helped rule out neurogenetic and infectious causes, making DS more likely. However, it is important to note that our patient's presentation was atypical in that the HBHC did not disappear with sleep; there are only two reported cases of DS that showed no suppression of HBHC during sleep [11,12]. 

The list of differential diagnoses can be further narrowed down by considering the causes of T1 hyperintensities of the striatum on the brain MRI. According to the biophysics of T1-weighted MRI sequences, T1-shortening leading to hyperintensities can be induced by melanin, methemoglobin in subacute hemorrhage, fat, slow-velocity blood flow, high protein content, and paramagnetic transition metals with unpaired electrons such as manganese, iron, zinc, and copper [13]. Clinical presentations of DS should help rule out some of these causes of T1-hyperintensity. For instance, copper and iron can be excluded as copper-deposition disorders, such as Wilson’s disease, and neuroferritinopathies, such as neurodegeneration associated with pantothenate-kinase, are chronic diseases with insidious onset [14]. Manganese can also be excluded as manganese-deposition diseases usually cause a Parkinsonian syndrome [15]. The already-established MRI characteristics of some substances can also aid in the process of elimination. Early subacute blood on an MRI brain demonstrates hypodensities on both susceptibility-weighted imaging (SWI) and apparent diffusion coefficient (ADC) sequences, and late subacute blood shows hyperintensity on diffusion-weighted imaging (DWI) sequences [16,17]. An ischemic infarct would show high signal intensity on DWI sequence and low signal intensity on ADC sequence [18]. Fat can easily be eliminated on fat-suppression images. Calcium, despite paired electrons, is paramagnetic as it is a metal; while it is dark on both SWI and gradient-recalled echo (GRE) sequences, it is bright on phase-filtered SWI sequences [19].

As can be seen, this two-fold approach will leave high protein content as the cause of the MRI T1-hyperintensity, which confirms the diagnosis of a true DS. The high protein density is accounted for by the gemistocytes involved in the pathophysiology of DS. Gemistocytes are swollen reactive astrocytes involved in acute brain injury, and their cytoplasm is enriched with a protein hydration layer which restricts the motion of water molecules by electrostatic repulsion, thereby shortening the T1-relaxation time [20].

## Conclusions

DS is a rare hyperkinetic movement disorder associated with poorly controlled diabetes and characterized by HBHC. The diagnosis of DS can be complicated by the many possible causes of HBHC. A two-fold approach that narrows down the possible diagnoses based on the patient’s clinical presentation and the brain MRI characteristics can be an effective tool for the diagnosis of DS. Future research endeavors should focus on establishing a standardized protocol for DS diagnosis.

## References

[1] Ondo WG (2011). Hyperglycemic nonketotic states and other metabolic imbalances. Handb Clin Neurol.

[2] Oh SH, Lee KY, Im JH, Lee MS (2002). Chorea associated with non-ketotic hyperglycemia and hyperintensity basal ganglia lesion on T1-weighted brain MRI study: a meta-analysis of 53 cases including four present cases. J Neurol Sci.

[3] Abe Y, Yamamoto T, Soeda T (2009). Diabetic striatal disease: clinical presentation, neuroimaging, and pathology. Intern Med.

[4] Cosentino C, Torres L, Nuñez Y, Suarez R, Velez M, Flores M (2016). Hemichorea/hemiballism associated with hyperglycemia: report of 20 cases. Tremor Other Hyperkinet Mov (N Y).

[5] Sitburana O, Ondo WG (2006). Tetrabenazine for hyperglycemic-induced hemichorea-hemiballismus. Mov Disord.

[6] Shan DE, Ho DM, Chang C, Pan HC, Teng MM (1998). Hemichorea-hemiballism: an explanation for MR signal changes. AJNR Am J Neuroradiol.

[7] Chua CB, Sun CK, Hsu CW, Tai YC, Liang CY, Tsai IT (2020). "Diabetic striatopathy": clinical presentations, controversy, pathogenesis, treatments, and outcomes. Sci Rep.

[8] Xie T, Awad I, Kang UJ, Warnke P (2014). DBS reduced hemichorea associated with a developmental venous anomaly and microbleeding in STN. Neurology.

[9] Tai CH, Pan MK, Tseng SH, Wang TR, Kuo CC (2020). Hyperpolarization of the subthalamic nucleus alleviates hyperkinetic movement disorders. Sci Rep.

[10] Rocha Cabrero F, De Jesus O (2021). Hemiballismus. https://www.ncbi.nlm.nih.gov/books/NBK559127/.

[11] Ruhangisa F, Stephen H, Senkondo J (2016). Acute hemichorea in a newly diagnosed type II diabetes patient: a diagnostic challenge in resource-limited setting: a case report. BMC Res Notes.

[12] Pinsker JE, Shalileh K, Rooks VJ, Pinsker RW (2015). Hemichorea-hemiballism secondary to non-ketotic hyperglycemia. J Clin Med Res.

[13] Ginat DT, Meyers SP (2012). Intracranial lesions with high signal intensity on T1-weighted MR images: differential diagnosis. Radiographics.

[14] Chang X, Zhang J, Jiang Y, Wang J, Wu Y (2020). Natural history and genotype-phenotype correlation of pantothenate kinase-associated neurodegeneration. CNS Neurosci Ther.

[15] Lucchini R, Placidi D, Cagna G, Fedrighi C, Oppini M, Peli M, Zoni S (2017). Manganese and developmental neurotoxicity. Adv Neurobiol.

[16] Heit JJ, Iv M, Wintermark M (2017). Imaging of intracranial hemorrhage. J Stroke.

[17] Khedr SA, Kassem HM, Hazzou AM, Awad E, Fouad MM (2013). MRI diffusion-weighted imaging in intracranial hemorrhage (ICH). Egypt J Radiol Nucl Med.

[18] Allen LM, Hasso AN, Handwerker J, Farid H (2012). Sequence-specific MR imaging findings that are useful in dating ischemic stroke. Radiographics.

[19] Wu Z, Mittal S, Kish K, Yu Y, Hu J, Haacke EM (2009). Identification of calcification with MRI using susceptibility-weighted imaging: a case study. J Magn Reson Imaging.

[20] Cherian A, Thomas B, Baheti NN, Chemmanam T, Kesavadas C (2009). Concepts and controversies in nonketotic hyperglycemia-induced hemichorea: further evidence from susceptibility-weighted MR imaging. J Magn Reson Imaging.

